# An In Vitro Calibration Model for Vancomycin Quantification in Brain Extracellular Fluid: Toward Improved Dosing in Postoperative Infections

**DOI:** 10.1002/prp2.70179

**Published:** 2025-10-14

**Authors:** Skaistė Žukaitienė, Ainė Žygaitė, Gabrielė Milkintaitė, Jolita Pancerė, Neringa Balčiūnienė, Astra Vitkauskienė, Romaldas Mačiulaitis

**Affiliations:** ^1^ Physiology and Pharmacology Institute, Medical Academy Lithuanian University of Health Sciences Kaunas Lithuania; ^2^ Medical Academy Lithuanian University of Health Sciences Kaunas Lithuania; ^3^ Hospital of Lithuanian University of Health Sciences Kauno Klinikos Kaunas Lithuania; ^4^ Department of Intensive Care, Medical Academy Lithuanian University of Health Sciences Kaunas Lithuania; ^5^ Department of Laboratory Medicine, Medical Academy Lithuanian University of Health Sciences Kaunas Lithuania

**Keywords:** calibration, central nervous system infections, microdialysis, pharmacokinetics, vancomycin

## Abstract

Postoperative central nervous system (CNS) infections are associated with high mortality and present a therapeutic challenge due to limited antibiotic penetration into the brain extracellular fluid (ECF). Vancomycin, frequently used in this setting, requires therapeutic drug monitoring; however, its quantification in brain ECF via microdialysis is limited by the need for accurate calibration of relative recovery (RR). This study aimed to determine the in vitro RR of vancomycin under conditions simulating clinical neuromonitoring to support application in clinical practice. Vancomycin RR was assessed using forward dialysis and retrodialysis techniques at a fixed perfusion rate of 0.3 μL/min, consistent with standard neuromonitoring protocols. Vancomycin concentrations were measured using a homogeneous enzyme immunoassay across subtherapeutic, therapeutic, and supratherapeutic levels. RR was calculated as the ratio of microdialysate to reference solution concentrations. Mean RR was 86.5% (SD 3.6%) for forward dialysis and 86.4% (SD 2.1%) for retrodialysis, with no significant difference between techniques (*p* = 0.957). RR remained consistent across all tested concentration levels (*p* = 0.051). A strong correlation was observed between vancomycin concentrations in the microdialysate and the study solution (*r* = 0.997, *p* < 0.001). The high and stable RR achieved under clinically relevant conditions supports the use of this in vitro microdialysis model as a reliable calibration tool. This model may aid in estimating vancomycin concentrations in brain fluid, facilitating dose optimization in patients undergoing microdialysis‐based monitoring for postoperative CNS infections.

## Graphical abstract

Overview of the study design, methodology, and main findings on vancomycin relative recovery using in vitro microdialysis under neurocritical care–simulated conditions.

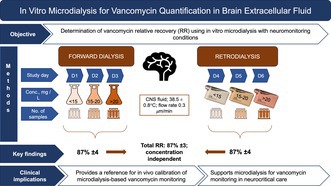

## 1 Introduction

Postoperative bacterial meningoencephalitis (POM) is a serious complication of neurosurgery, associated with high mortality rates ranging from 5% to 47.7%, significant neurological deficits, prolonged hospital stays, and increased overall healthcare costs [1, 2, 3, 4, 5, 6]. Prognosis depends on several factors, including the timeliness of diagnosis, virulence of the causative pathogen, underlying medical conditions, patient age, neurological status, and the appropriateness of the selected antibiotic (AB) regimen [7]. While many of these determinants are non‐modifiable, optimizing those that are—particularly ensuring adequate antibiotic concentrations at the site of infection—is crucial for improving patient outcomes.

Although plasma drug monitoring remains standard practice [8], plasma concentrations often do not accurately reflect drug levels at the actual site of infection, which in the case of bacterial meningitis or meningoencephalitis is the brain extracellular fluid (ECF) [9, 10, 11]. Achieving therapeutic concentrations in the brain is further complicated by the restrictive properties of the blood–brain barrier (BBB) [12, 13, 14, 15]. As such, optimizing antimicrobial efficacy requires attainment of well‐defined pharmacokinetic/pharmacodynamic (PK/PD) targets directly at the site of infection [16, 17, 18]. The ability to assess PK/PD target attainment within the brain ECF could significantly enhance clinical decision‐making and treatment effectiveness.

In this context, microdialysis (MD) offers a promising technique for real‐time monitoring of drug levels, provided that methodological limitations—particularly those related to relative recovery (RR)—are effectively addressed. MD is an advanced sampling technique that enables continuous measurement of both endogenous and exogenous compounds in the ECF [19]. Cerebral MD is currently the only method capable of direct sampling of brain ECF and is routinely employed in neurocritical care for monitoring cerebral metabolism in patients with severe traumatic brain injury or poor‐grade subarachnoid hemorrhage [20, 21].

Despite its established role in neuromonitoring, MD is not yet routinely used in clinical practice to quantify drug concentrations in brain ECF. This is largely due to methodological challenges, such as the need for precise calibration and the difficulty of translating research protocols into standardized clinical workflows. A critical component of MD calibration is the RR, which represents the proportion of a substance recovered in the dialysate relative to its actual concentration in the surrounding medium [19, 21]. While in vivo recovery techniques are considered the gold standard, their implementation is often limited by practical constraints.

RR is influenced by multiple factors, including perfusate flow rate, temperature, membrane characteristics, probe geometry, membrane surface area, tubing configuration, perfusate composition, and the physicochemical properties of the drug itself [19, 22]. Furthermore, pathological states such as inflammation, tumor presence, or traumatic brain injury may lead to additional variability in recovery, causing regional differences in drug concentrations [19]. Addressing these variables is essential to improve the reliability and accuracy of MD‐based drug quantification.

Overcoming these challenges could enable MD to serve as a powerful tool for quantifying vancomycin concentrations in brain ECF, especially in neurosurgical patients with bacterial meningitis or meningoencephalitis who are already undergoing routine multimodal neuromonitoring. Accurate determination of vancomycin levels in brain ECF would support individualized dose optimization, ensuring therapeutic efficacy while minimizing toxicity risk.

Vancomycin—a glycopeptide antibiotic—is recommended by the Infectious Diseases Society of America (IDSA) for empirical treatment of healthcare‐associated ventriculitis and meningitis. It is frequently administered alongside an anti‐pseudomonal beta‐lactam and remains the first‐line therapy for infections caused by methicillin‐resistant *Staphylococcus aureus* (MRSA) and other β‐lactam‐resistant Gram‐positive bacteria [23]. However, vancomycin's high molecular weight (~1450 Da) and hydrophilic nature limit its ability to cross the BBB, especially in the absence of meningeal inflammation [24]. Although several studies have reported limited cerebrospinal fluid (CSF) penetration [12, 13], data on vancomycin distribution specifically within brain ECF—the actual infection site in neurosurgical cases—are lacking. The only study to date investigating vancomycin penetration into brain ECF, conducted by Caricato et al. [25], has been criticized for failing to account for RR correction, which is essential for obtaining accurate concentration measurements [26].

This study aimed to determine the RR of vancomycin using in vitro MD under conditions that replicate in vivo neuromonitoring practices in a tertiary hospital setting. By employing the same MD pump, catheter, perfusion fluid, and analytical methods used in clinical care, the goal was to develop a robust model capable of accurately estimating vancomycin concentrations in brain ECF during routine MD‐based neuromonitoring. Such a model could enable real‐time therapeutic drug monitoring and individualized dose adjustments, thereby enhancing treatment outcomes in neurocritical care patients.

## 2 Methods

### 2.1 Chemicals and Preparation

**FIGURE 1:**
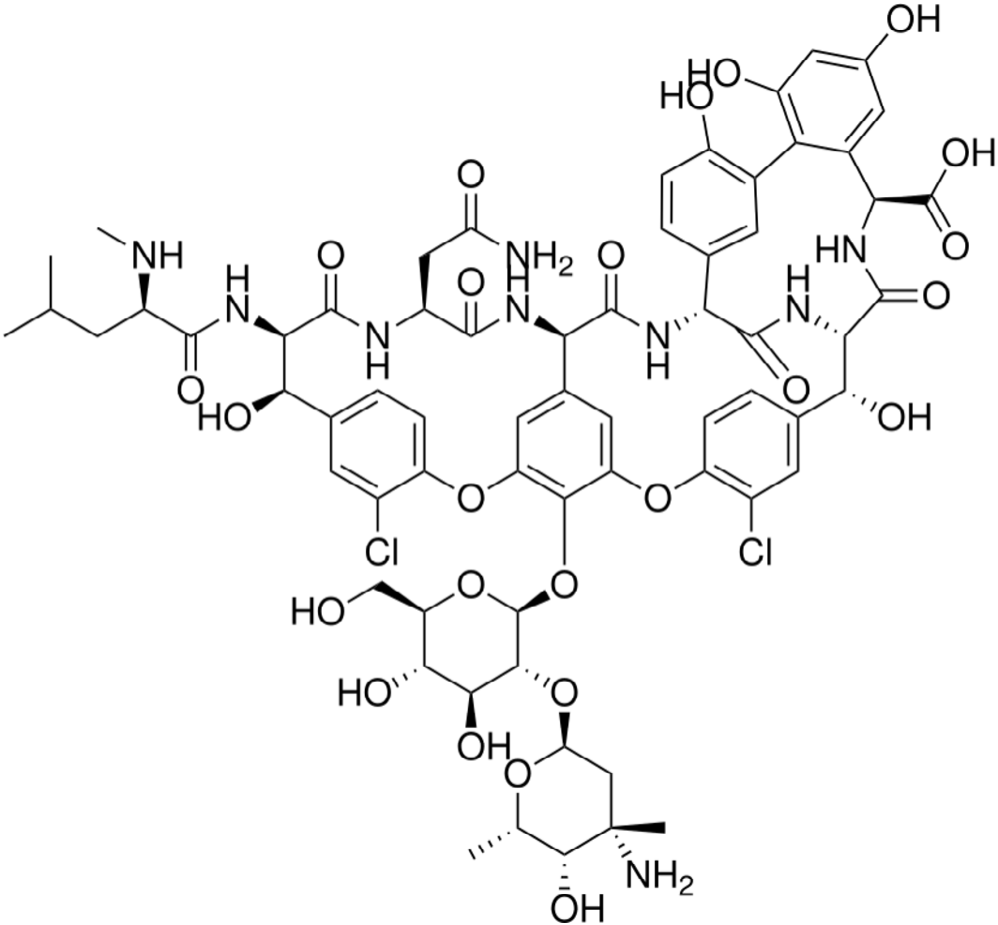
Structure of vancomycin.

Chromatographically purified vancomycin hydrochloride (Vancomax 500 mg, John Lee Pharmaceuticals Ltd.) was obtained from the hospital pharmacy, where it was routinely used in clinical practice at the time of the study. Vancomycin was supplied as a powder for concentrate for solution for infusion. Initially, the powder was dissolved in 10 mL of sterile water for injection, yielding a stock solution with a vancomycin concentration of 50 mg/mL. Prior to further dilution, the solution was visually inspected to confirm its clarity and absence of particulate matter, with a color range from colorless to slightly yellowish‐brown, in accordance with standard quality control criteria. The chemical structure of vancomycin is shown in Figure 1.

The reconstituted stock solution was subsequently diluted with Central Nervous System (CNS) perfusion fluid (M Dialysis AB, Sweden) to prepare the immersion solution, which mimicked extracellular brain fluid, for the forward dialysis (FWD) experiment or the perfusion solution for the retrodialysis (RD) experiment, depending on the study protocol. The CNS perfusion fluid, an isotonic sterile solution specifically designed for microdialysis in brain tissue, contained 147 mmol/L of sodium chloride, 2.7 mmol/L of potassium chloride, 1.2 mmol/L of calcium chloride, and 0.85 mmol/L of magnesium chloride. The total chloride content was 153.8 mmol/L, with an osmolality of 305 mOsm/kg and a pH of approximately 6. This perfusion fluid was used as the immersion and perfusion solution throughout all experiments.

### 2.2 Microdialysis System

The microdialysis system included a 70 Microdialysis Bolt Catheter (M Dialysis AB, Stockholm, Sweden) with a shaft composed of polyurethane, measuring 130 mm in length and 0.9 mm in diameter. The catheter membrane was made of polyarylethersulfone (PAES) with a length of 10 mm and a diameter of 0.6 mm. The inlet tubing had an internal diameter of 0.15 mm and an outer diameter of 1.0 mm, while the outlet tubing had an internal diameter of 0.15 mm and an outer diameter of 0.9 mm. The membrane had a molecular weight cut‐off of 20 000 Da, ensuring selective diffusion of vancomycin while minimizing interference from larger molecules.

The 106 MD Pump (M Dialysis AB, Sweden) was used to deliver a continuous perfusion flow at a fixed rate of 0.3 μL/min. During the initial priming phase, the pump operated at a higher flow rate of 15 μL/min for the first 5 min to ensure complete system filling before automatically adjusting to the experimental flow rate of 0.3 μL/min. Dialysates were collected in microvials with a total capacity of 200 μL, ensuring an adequate volume for subsequent analysis.

### 2.3 Experimental Setup

Two microdialysis techniques were employed to evaluate vancomycin relative recovery: FWD and RD. In the FWD experiments, the reconstituted vancomycin stock solution was diluted with CNS perfusion fluid to achieve predefined concentration levels. The microdialysis pump syringe was filled with standard CNS perfusion fluid, and the concentration of vancomycin recovered in the dialysate was measured to determine RR. In the RD experiments, vancomycin was directly introduced into the perfusate at known concentration levels, while the immersion fluid consisted of CNS perfusion fluid without vancomycin. The amount of vancomycin diffusing out of the probe into the immersion fluid was quantified to calculate relative recovery.

RR was assessed at three predefined concentration levels representing subtherapeutic, therapeutic, and supratherapeutic ranges. The subtherapeutic range included concentrations below 15 mg/L (10.3 μmol/L), the therapeutic range was defined as concentrations between 15 and 20 mg/L (10.3 and 13.8 μmol/L), and the supratherapeutic range included concentrations exceeding 20 mg/L (13.8 μmol/L).

To determine the actual vancomycin concentration in the prepared study solutions and assess their stability and potential evaporation effects, vancomycin concentrations were measured at three time points: before the experiment to establish baseline values, at the midpoint of the experiment approximately 6 h after initiation, and at the conclusion of the experiment.

To ensure a homogenous temperature and concentration environment, a magnetic stirring bar on a hotplate stirrer was used to constantly mix the solution at 300 rpm during the experiment. This stirring rate was selected based on visual inspection (i.e., ensuring gentle circulation without vortex formation or probe displacement) and is consistent with values reported in the literature for in vitro microdialysis calibration experiments [27, 28]. The temperature was controlled at 38.5°C ± 0.8°C, corresponding to the mean brain temperature, as described in previous studies [29]. The temperature of the study solutions was continuously recorded and adjusted as necessary to maintain experimental consistency.

The study was conducted over six consecutive days, with 3 days allocated to forward dialysis experiments and 3 days to retrodialysis experiments. To eliminate potential residual vancomycin adherence that could affect relative recovery calculations, separate microdialysis catheters were used for each experiment. The experiments were initiated with subtherapeutic concentrations and progressively increased to supratherapeutic levels to minimize potential carryover effects. Throughout all experiments, the solutions were kept under continuous agitation using a magnetic stirrer to ensure homogenization, and temperature was precisely regulated to mimic in vivo conditions.

Each experiment, including forward dialysis and retrodialysis, was conducted over 3 days, with each day allocated to a specific concentration level: subtherapeutic, therapeutic, or supratherapeutic. On each experimental day, three microdialysate samples were collected, resulting in a total of 18 samples across both experiments. The collection of each microdialysate sample required a duration of 4 h, ensuring sufficient volume for accurate analysis. The first microdialysate samples collected during the initial hour of each experiment were discarded due to the elevated flow rate (15 μL/min) used during the priming phase. Each microdialysate sample had a volume of approximately 75 μL, which was sufficient for accurate concentration analysis. Immediately upon collection, the samples were analyzed for vancomycin concentration to ensure precise determination of relative recovery.

### 2.4 Analytical Method

Vancomycin concentrations in microdialysate samples and prepared study solutions were determined using the Emit 2000 Vancomycin Assay, a homogeneous enzyme immunoassay routinely employed in the clinical laboratory of the hospital where the study was conducted. This method was selected due to its rapid turnaround time and high specificity, making it suitable for both clinical settings and pharmacokinetic research involving vancomycin.

The Emit 2000 Vancomycin Assay operates on a competitive inhibition immunoassay principle. Vancomycin in the sample competes with enzyme‐labeled vancomycin (conjugated with glucose‐6‐phosphate dehydrogenase, G6PDH) for binding sites on specific antibodies. In the presence of vancomycin, less enzyme‐labeled conjugate binds to the antibody, leading to increased enzyme activity. The active G6PDH enzyme catalyzes the conversion of oxidized nicotinamide adenine dinucleotide (NAD) to its reduced form (NADH), causing a measurable change in absorbance. This change is detected spectrophotometrically and is directly proportional to the vancomycin concentration in the sample.

All assays were conducted using a Beckman Coulter AU680 analyzer, following the manufacturer's protocol. Although the method is primarily validated for human plasma and serum, it was successfully adapted in this study for use with artificial perfusion fluid (Perfusion Fluid CNS, M Dialysis AB, Sweden) and collected microdialysates, in line with the in vitro experimental design.

Calibration was performed using commercial vancomycin calibrators (Emit 2000 Vancomycin Calibrators, Siemens Healthcare Diagnostics) within the range of 2–50 mg/L, according to the instrument operator's manual. These calibrators are supplied in a human serum matrix, which serves as the solvent and supports the standardization of assay response under routine clinical use. Multi‐level commercial quality control materials with known vancomycin concentrations were included in each run to confirm the accuracy and reliability of the calibration curve.

To assess potential matrix interference from the perfusate and microdialysate samples, an internal matrix effect evaluation was conducted. Drug‐free perfusion fluid (Perfusion Fluid CNS) was spiked with vancomycin at three concentration levels (low, medium, and high within the assay range). These spiked samples were analyzed in parallel with serum‐based calibrators. Analytical recovery was calculated by comparing measured versus nominal concentrations, and results were considered acceptable if within 85%–115% of the nominal value. Additionally, visual inspection of calibration curve slope and linearity suggested that assay response in the artificial matrix was comparable to that observed in the serum‐based calibrators. Coefficients of variation (CV%) for replicate measurements remained below 15%, indicating consistent performance. Based on these findings, no significant matrix effect was observed, and the assay was deemed appropriate for quantifying vancomycin in microdialysate under the conditions of this study.

### 2.5 Data Analysis

RR was calculated according to the following equations:

$\mathrm{RR}\left(\%\right)=\left(\frac{C_{\text{dial}}}{C_{\mathrm{imm}}}\right)\times 100$ for FWD, where *C*_dial_ is the concentration of vancomycin in the microdialysate, and *C*_imm_ is the concentration in the immersion solution.

$\mathrm{RR}\left(\%\right)=\left(\frac{C_{\text{perf}}-C_{\text{dial}}}{C_{\text{perf}}}\right)\times 100$ for RD, where *C*_dial_ is the concentration of vancomycin in the microdialysate, and *C*_perf_ is the concentration in the perfusate.

Descriptive statistics (mean, standard deviation, minimum, and maximum) were used. An independent‐samples *t*‐test was conducted to assess differences in RR between the two techniques, and a one‐way ANOVA was used to evaluate RR across concentration ranges. Pearson's correlation and linear regression analyses were conducted to evaluate relationships between measured and prepared vancomycin concentrations. A *p*‐value < 0.05 was considered statistically significant.

All statistical analyses were performed using IBM SPSS Statistics version 30.0.0.0 (172).

## 3 Results

### 3.1 Experimental Adjustments and Sample Allocation

A total of 18 microdialysate samples were collected as planned. However, higher‐than‐expected evaporation effects during the FWD experiment led to an imbalance in sample distribution across the predefined vancomycin concentration levels. According to the study protocol, three samples were to be collected at each level: subtherapeutic, therapeutic, and supratherapeutic. While all three concentration ranges were captured, only two samples represented the therapeutic level (instead of the intended three), whereas three and four samples were collected in the subtherapeutic and supratherapeutic ranges, respectively. In contrast, all three concentration levels were successfully maintained in the RD experiment. For the purposes of RR calculation in the FWD group, vancomycin concentrations in the study solution were extrapolated using a linear regression model that estimated the average concentration during microdialysate collection. Specifically, the concentration measured prior to sample collection and an extrapolated value at the end of the collection period were averaged to represent the relevant concentration window. In the RD experiment, the actual vancomycin concentration measured in the study solution (perfusate) was used directly for RR calculation.

### 3.2 Recovery Rates Across Microdialysis Techniques

RR values for both microdialysis techniques were comparable. The FWD technique demonstrated a mean RR of 86.5% (SD 3.6%), while the RD technique exhibited a mean RR of 86.4% (SD 2.1%) (Table 1). An independent‐samples t‐test was conducted to compare the RR between the two techniques, revealing no statistically significant difference, *t* (16) = 0.055, *p* = 0.957. This result indicates that both techniques performed similarly in terms of RR. The overall RR across both techniques was 86.4% (SD 2.8%), with observed values ranging from 81.9% to 91.7%.

**TABLE 1:** Relative recovery (RR) based on microdialysis technique.

Technique	Mean RR (SD), %	Min–Max, %
FWD	86.5 (3.6)	81.9–91.2
RD	86.4 (2.1)	82.7–89.2

### 3.3 Recovery Rates Across Therapeutic Levels

The RR rates across subtherapeutic, therapeutic, and supratherapeutic concentration levels were consistent, with no statistically significant variations. The mean RR values were 88.2% (SD 1.2%) for subtherapeutic, 84.2% (SD 2.1%) for therapeutic, and 86.5% (SD 3.4%) for supratherapeutic levels (Table 2). A one‐way ANOVA test suggested the absence of significant differences among these groups (*F* (2, 15) = 3.660, *p* = 0.051).

**TABLE 2:** Recovery rates across therapeutic levels.

Concentration level	Mean RR (SD), %	Min–Max, %
Subtherapeutic	88.2 (1.2)	86.8–89.9
Therapeutic	84.2 (2.1)	81.9–86.7
Supratherapeutic	86.5 (3.4)	82.3–91.7

### 3.4 Correlation Between Prepared Study Solution and Microdialysate

**FIGURE 2:**
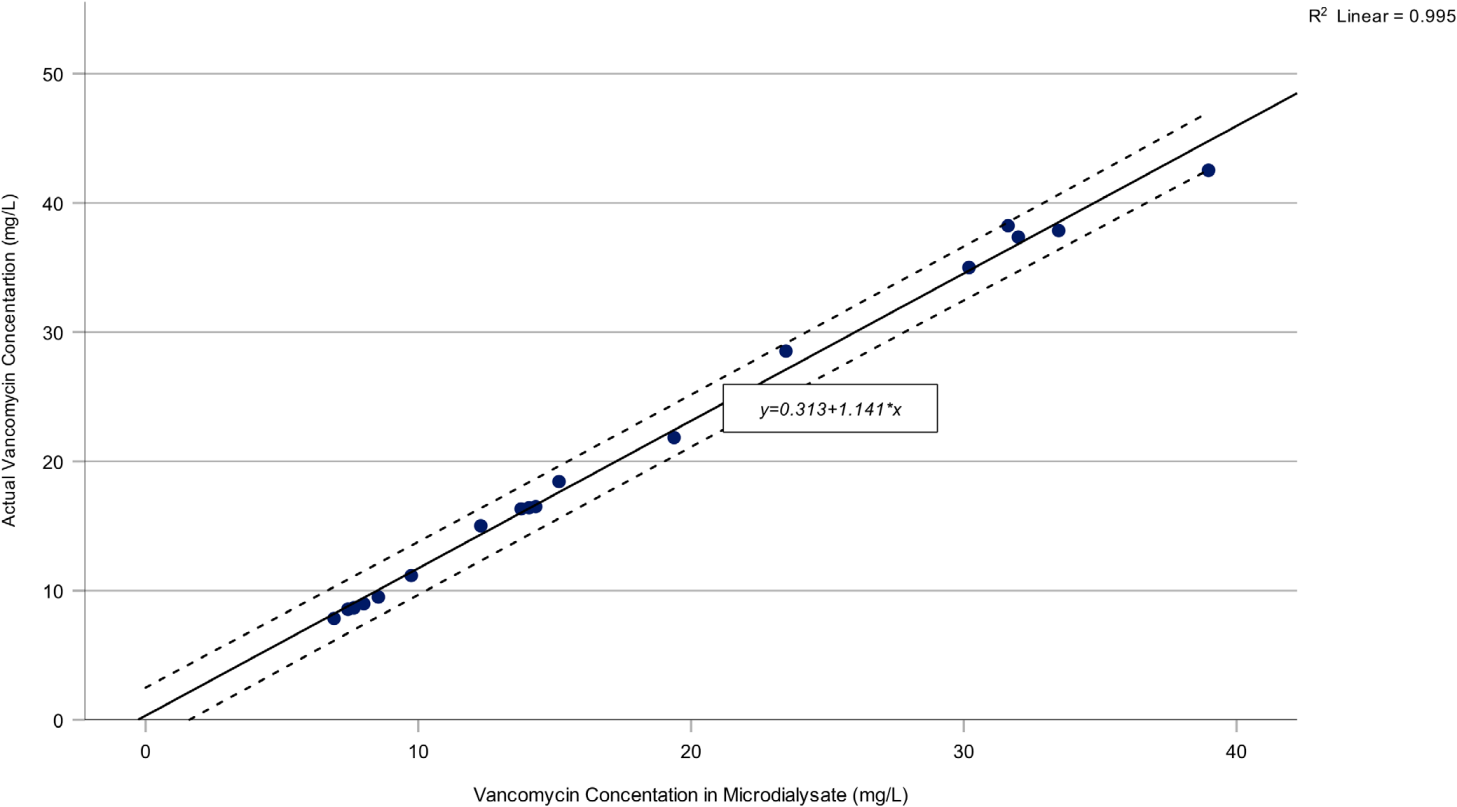
Correlation between Vancomycin Concentration in the Study Solution and Microdialysate. The scatter plot illustrates the linear relationship between vancomycin concentrations measured in the microdialysate and those in the corresponding study solution. The regression equation shown represents the predictive model used to estimate the vancomycin concentration in the study solution based on microdialysate values.

**FIGURE 3:**
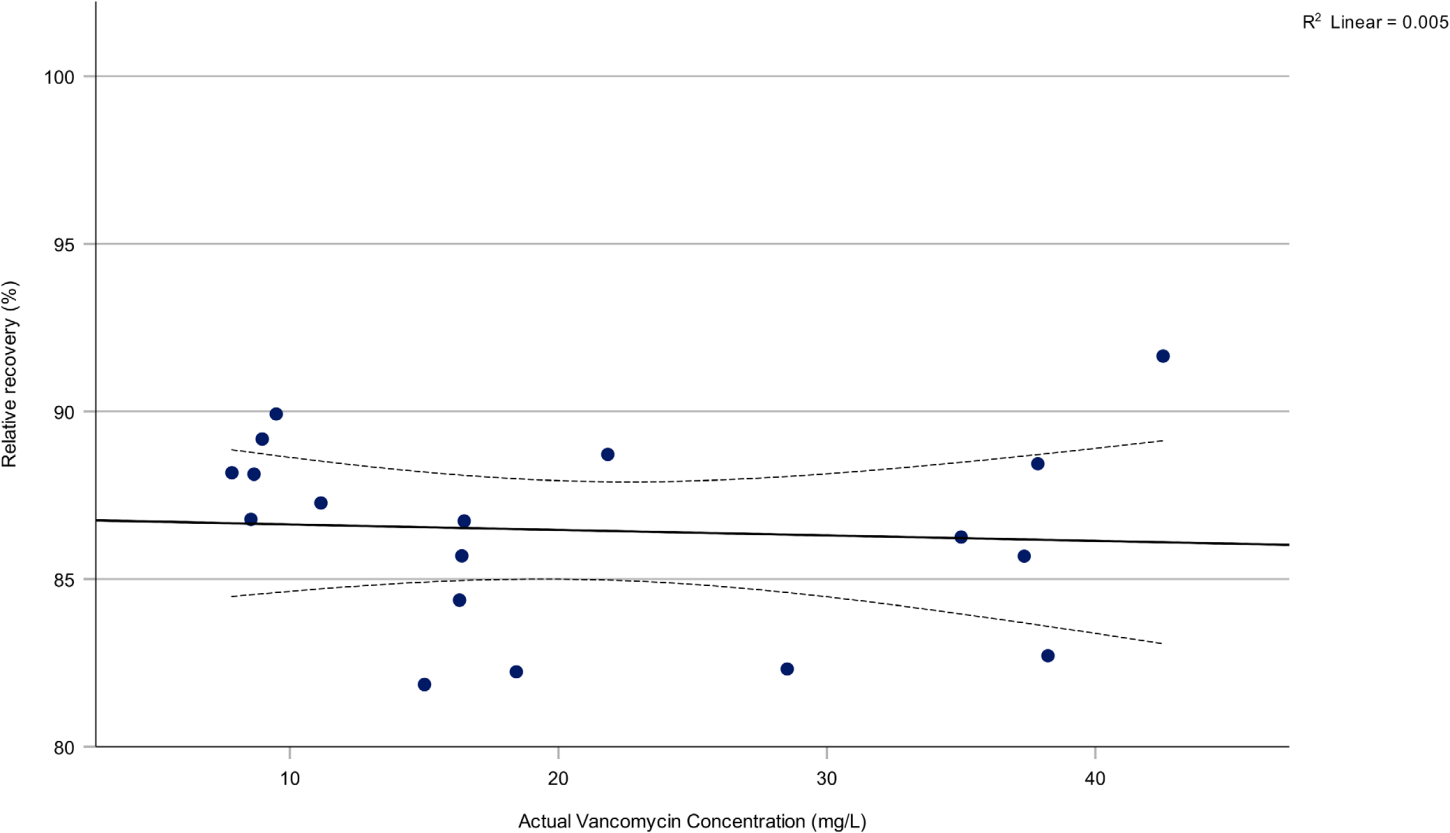
Relationship between vancomycin concentration in the study solution and relative recovery (RR). Scatter plot showing the correlation between actual vancomycin concentrations in the prepared study solution and corresponding RR values. No statistically significant correlation was found (*r* = −0.070, *p* = 0.782), indicating that relative recovery remained stable across the range of tested concentrations.

A strong positive correlation was observed between vancomycin concentration in the prepared study solution and microdialysate. Pearson's correlation coefficient was *r* = 0.997, *p* < 0.001, indicating a robust linear association between the two variables (Figure 2). In contrast, no statistically significant correlation was detected between actual vancomycin concentration in the study solution and relative recovery RR (*r* = −0.070, *p* = 0.782), suggesting that RR remained stable regardless of drug concentration (Figure 3).

### 3.5 Regression Analysis

A linear regression analysis was performed to model the relationship between vancomycin concentration in the microdialysate and prepared study solution (Figure 2). The regression equation was derived as follows:

y=0.313+1.141×x

where *y* denotes the actual vancomycin concentration in the prepared study solution, and *x* represents the vancomycin concentration recovered in the microdialysate.

The model demonstrated excellent predictive accuracy, explaining 99.5% of the variance in vancomycin concentration (adjusted *R* ^2^ = 0.995, *p* < 0.001). The regression analysis indicated that for every 1 mg/L increase in vancomycin concentration in microdialysate, the actual vancomycin concentration in study solution increased by 1.141 mg/L (95% CI: 1.096–1.186 mg/L). A scatterplot with a superimposed regression line confirmed a linear relationship between the two variables, with no heteroscedasticity or outliers identified. These findings support the reliability of in vitro microdialysis recovery data for in vivo calibration.

## 4 Discussion

The present study demonstrated that the in vitro microdialysis recovery of vancomycin under conditions mimicking clinical neuromonitoring yielded a high RR of approximately 86%. This indicates that microdialysis is a feasible method for assessing vancomycin distribution in brain ECF, particularly when flow rates are set at 0.3 μL/min, a parameter that aligns with standard neuromonitoring practices. This flow rate is widely used in neurocritical care settings as part of cerebral microdialysis protocols, as it provides a balance between adequate analyte recovery and minimal disturbance to the extracellular environment [20]. The significance of this study lies in its potential application in clinical practice, where in vitro recovery data can be utilized to guide therapeutic decisions. While precise pharmacokinetic data are essential in PKPD studies for drug development, such accuracy is of less importance in clinical practice, where broader pharmacokinetic principles and patient response play a more significant role.

A notable limitation of this study is the absence of direct comparative data from previous research employing identical or closely similar experimental conditions. While prior investigations have explored vancomycin recovery using in vitro microdialysis, methodological differences—particularly in flow rate settings—complicate direct comparisons. Studies examining recovery rates at higher flow rates have demonstrated a proportional decrease in RR, while research conducted at lower flow rates has reported higher RR values, reinforcing the inverse relationship between perfusate flow rate and RR [30, 31]. These findings underscore the importance of standardizing microdialysis conditions to ensure reliable inter‐study comparisons and enhance applicability for clinical practice and research purposes.

Interestingly, when applying a previously proposed equation [30] to the conditions of our study (0.3 μL/min flow rate), the predicted RR would be approximately 99%, which substantially overestimates the experimentally determined RR of 86%, suggesting that theoretical models may not fully account for factors affecting RR at extremely low flow rates and highlighting the necessity of empirical validation.

A critical limitation of in vitro microdialysis is its inability to directly translate recovery rates to in vivo conditions. In vitro recoveries have been traditionally used to estimate in vivo extracellular concentrations, but this approach has significant limitations and does not fully account for the complexities of in vivo conditions. In vivo microdialysis recovery is influenced by additional physiological factors such as tissue tortuosity, capillary exchange, cellular uptake, metabolic degradation, active transport, and local blood flow [22]. Given these complexities, in vitro RR values should not be interchangeably applied to in vivo settings without proper calibration. Comparative studies between in vitro and in vivo microdialysis methods have not demonstrated a significant correlation [19, 32, 33], further emphasizing the need for in vivo validation.

Another limitation worth noting is that the potential adsorption of vancomycin to the microdialysis tubing (polyurethane) and membrane (polyarylethersulphone, PAES) was not experimentally evaluated. Although adsorption is a known concern in microdialysis—especially for lipophilic or highly protein‐bound drugs—vancomycin is a hydrophilic compound with moderate protein binding (~50%), reducing its likelihood of nonspecific binding. In our study, all microdialysate samples contained vancomycin concentrations above the lower limit of quantification, and the average relative recovery was high (86%), suggesting that adsorption did not significantly affect the measurements. Moreover, published data indicate low adsorption of vancomycin to PAES (recovery 87.8%–90.5%) and polyurethane (recovery > 96.3%) in other clinical and experimental contexts [34, 35]. Nonetheless, because adsorption‐related losses cannot be fully ruled out, future studies should incorporate pretesting or correction for potential analyte loss due to binding to system components.

Despite these limitations, the findings of this study provide a valuable approximation of vancomycin RR under controlled conditions, which may be useful in calibrating in vivo microdialysis studies. Notably, there are no existing studies directly comparing in vitro and in vivo vancomycin RR in patients with post‐neurosurgical CNS infections. The RR values obtained in this study could serve as an initial reference point for determining vancomycin concentrations in brain ECF in clinical practice.

In clinical applications, understanding that vancomycin concentrations in microdialysate represent a fraction of the true ECF concentration is of paramount importance. If microdialysate concentrations are within the therapeutic range (15–20 mg/L) at the end of a dosing interval or during continuous infusion, clinicians can reasonably infer that CNS exposure is adequate without adjusting for RR. Conversely, if microdialysate concentrations fall below the therapeutic threshold, applying the best available RR estimates is necessary to guide dosing adjustments. For instance, if an in vivo microdialysis study measured vancomycin at 13 mg/L, adjusting for an 86% RR would yield an estimated true ECF concentration of 15.93 mg/L, suggesting therapeutic adequacy.

It is important to note that in microdialysis, the measured concentration represents the average over the sampling interval (in this case, 4 h), rather than a discrete trough or peak. Therefore, the setup does not allow for precise determination of time‐specific concentrations such as trough levels. However, for vancomycin, trough concentrations are less clinically relevant than the overall AUC_0–24_ [8], which remains interpretable if sampling avoids the initial distribution phase. Furthermore, as vancomycin is frequently administered via continuous infusion in clinical practice—including in our center—the concept of a trough becomes less meaningful, and steady‐state or average exposure is of greater importance.

Given the likelihood that in vivo RR values are lower than their in vitro counterparts due to additional diffusion barriers, the true ECF concentration may be even higher than estimated. This underscores the need for further research to refine RR estimations and validate their clinical applicability. Future studies should focus on direct in vivo–in vitro comparisons of vancomycin RR in patients undergoing multimodal neuromonitoring for CNS infections.

We encourage clinicians and researchers working in neurocritical care to adopt the in vitro recovery model proposed in this study as a practical tool for estimating vancomycin concentrations in brain ECF. This model is particularly applicable to patients with postoperative CNS infections who are already undergoing cerebral microdialysis as part of routine multimodal neuromonitoring. By incorporating RR‐based calibration into their monitoring protocols, teams can improve real‐time therapeutic assessment and inform individualized dosing strategies, ultimately contributing to better clinical outcomes in this high‐risk population.

## 5 Conclusion

Vancomycin relative recovery, as determined by in vitro microdialysis, may serve as a practical surrogate for in vivo RR when experimental conditions closely mirror those used in clinical neuromonitoring. Although not a replacement for in vivo studies, this model offers a useful approximation to guide therapeutic decisions in patients with postoperative CNS infections. We invite clinicians and researchers in neurocritical care to apply this model in both clinical practice and applied research to support individualized vancomycin dosing based on brain ECF concentrations.

## Author Contributions

**Skaistė Žukaitienė:** conceptualization, data curation, formal analysis, funding acquisition, investigation, methodology, project administration, visualization, writing – original draft, writing – review and editing. **Ainė Žygaitė:** investigation, visualization, writing – original draft. **Gabrielė Milkintaitė:** visualization, writing – original draft. **Jolita Pancerė:** investigation, writing – review and editing. **Neringa Balčiūnienė:** resources, writing – review and editing. **Astra Vitkauskienė:** resources, validation, writing – review and editing. **Romaldas Mačiulaitis:** conceptualization, methodology, writing – review and editing.

## Disclosure

Declaration of Generative AI Use: During the preparation of this work, the authors used ChatGPT by OpenAI in order to improve readability and language. After using this tool, the authors reviewed and edited the content as needed and took full responsibility for the content of the publication.

## Ethics Statement

The authors have nothing to report.

## Conflicts of Interest

The authors declare no conflicts of interest.

## Data Availability

The data that support the findings of this study are available from the corresponding author upon reasonable request.
